# The Role of Dermoscopy and High-Frequency Ultrasonography in the Diagnosis and Monitoring of Psoriasis Vulgaris

**DOI:** 10.3390/medicina61111978

**Published:** 2025-11-04

**Authors:** Ioana-Alina Halip, Dan Vata, Adriana Ionela Patrascu, Doinita Temelie Olinici, Ioana-Adriana Popescu, Madalina Mocanu, Dumitrita Lenuta Gugulus, Valentin-Tudor Popa, Dragos Gheuca-Solovastru, Laura Gheuca-Solovastru

**Affiliations:** 1 Department of Dermatology, Grigore T. Popa University of Medicine and Pharmacy, 700115 Iasi, Romania; alinaioanahalip@gmail.com (I.-A.H.); oana.manolache@yahoo.com (I.-A.P.); drmadalinamocanu@yahoo.com (M.M.); nichitean.dumi@yahoo.com (D.L.G.); lsolovastru13@yahoo.com (L.G.-S.); 2 Dermatology Clinic, “St. Spiridon” County Emergency Clinical Hospital, 700115 Iasi, Romania; patrascuai@yahoo.com (A.I.P.); doinitzaganceanu@yahoo.com (D.T.O.); 3 Department of Morpho-Functional Sciences II, Grigore T. Popa University of Medicine and Pharmacy, 700115 Iasi, Romania; 4 Anapatmol Research Center, “Victor Babeș” University of Medicine and Pharmacy, 300041 Timișoara, Romania; popa.valentin@umft.ro; 5 Faculty of Medicine, Grigore T. Popa University of Medicine and Pharmacy, 700115 Iasi, Romania; dragosflorin13@yahoo.com

**Keywords:** dermoscopy, high-frequency-ultrasound examination, psoriasis vulgaris, therapeutic response

## Abstract

Psoriasis vulgaris is a polygenic, immunomediated dermatological condition, characterized pathophysiologically by abnormal proliferation of the epidermis and immune response disorders, evidenced by the presence of a dermal inflammatory infiltrate accompanied by exocytosis. The prevalence of this disease is continuously increasing, and the significant impact on quality of life is determined by both the severity of the skin manifestations and the associated comorbidities, which underlines the importance of early diagnosis. Among the imaging methods useful in the diagnosis and monitoring of psoriasis vulgaris are dermatoscopy and high-frequency cutaneous ultrasonography (HFUS). Dermatoscopy is a valuable complementary imaging tool in assessing the therapeutic response in patients with psoriasis vulgaris. Although clinical lesions may show partial or complete remission, the persistence of the specific vascular architecture—characterized by dilated and branched capillaries—suggests the maintenance of disease activity and justifies the need for continued treatment. HFUS allows the identification of characteristic changes in psoriatic plaques, such as homogeneous thickening of the epidermis, visible as a hyperechoic band, the presence of a hypoechoic subepidermal band, and thickening of the dermis. Evaluation with 20 MHz probes can significantly contribute to monitoring therapeutic efficacy, since the first observable changes under topical and/or systemic treatment include a reduction in the thickness of the epidermis, dermis, and hypoechoic subepidermal band. The integration of dermatoscopy and HFUS within the clinical evaluation allows for a complex and precise approach to the management of patients with psoriasis vulgaris, facilitating objective monitoring of disease progression and appropriate adjustment of therapy.

## 1. Introduction

Psoriasis vulgaris is a chronic, genetically determined, inflammatory and proliferative dermatosis clinically manifested by well-demarcated erythematous-squamous plaques that can affect the quality of life of patients. Psoriasis is associated with low self-esteem, anxiety (30%) and depressive disorders (60%) [1]. Treatment of psoriasis may contribute to the improvement of depressive symptoms, both by reducing the impact of psychodynamic factors associated with the disease and by decreasing levels of tumor necrosis factor alpha (TNF-alpha), which is involved in the pathogenesis of systemic inflammation and affective disorders. These aspects should be taken into account in the decision-making process regarding the individualized therapeutic strategy [2]. Considering the multitude of potential comorbidities associated with psoriasis vulgaris, optimal patient management requires a holistic, patient-centered approach and interdisciplinary collaboration between the specialties involved, in order to ensure integrated and effective treatment [3].

The clinical form of plaque psoriasis is characterized by the presence of well-demarcated erythematous-scaly skin lesions, varying in color from salmon pink to intense, shiny red. Thick, multilayered, white or silvery, non-adherent scales cover these plaques. The application of the Brocq method, by mechanical scraping, highlights the whitening and fragmentation of the scales, and the removal of the last layer can cause the appearance of a characteristic punctate bleeding (Auspitz sign), explained by the phenomenon of papillomatosis. The central resolution of the plaques can generate annular or arcuate aspects, while the confluence of small lesions can lead to the formation of areas with a “geographical map” appearance. The peripheral extension of the plaques can cause a circular or gyrated appearance. Preferential locations of plaque psoriasis vulgaris include the extensor areas of the limbs, especially the knees and elbows, the lumbosacral region, the scalp and the periumbilical area.

Practical and non-invasive methods are needed to monitor the progression and efficacy of plaque psoriasis treatment. The imaging techniques available for the assessment of psoriasis differ in terms of resolution, depth of penetration and visual representation.

The ability of dermatoscopy to highlight vascular details and subtle chromatic variations, difficult to perceive with the naked eye, explains its increasingly important role in the diagnosis of skin lesions. It finds extensive applications not only in general dermatology, but also in the evaluation of skin infections and infestations (entodermoscopy), hair and scalp disorders (trichoscopy), as well as in the diagnosis of periungual capillary abnormalities and inflammatory skin conditions [4].

Dermatoscopy of inflammatory conditions, such as psoriasis vulgaris, allows for detailed assessment of the vascular pattern, characterized by the presence of “dotted”, “pinpoint”, “coiled” capillaries, distributed uniformly, homogeneously or annularly on an erythematous background, accompanied by diffuse white scales. These dermatoscopic features have been documented in the literature as having a high diagnostic value, with a specificity of 88% and a sensitivity of 84.9% [5].

HFUS provides characteristic information that cannot be obtained by clinical examination and that can be integrated for a complex assessment of psoriasis vulgaris plaques, facilitating the assessment of disease activity and monitoring of the response to topical and/or systemic treatment. In plaque psoriasis lesions, thickening of the epidermis and dermis is observed, secondary to keratinocyte proliferation and inflammatory infiltrate. The epidermis shows a homogeneous, hyperechoic thickening, accompanied by a hypoechoic subepidermal band, and the dermis appears thickened and hypoechoic compared to the perilesional tissue, associated with increased blood flow. The hypoechoic subepidermal band reflects the presence of edema and inflammatory infiltrate in the papillary dermis. At the same time, the total thickness of the skin in psoriatic plaques is significantly increased, and its decrease under treatment can be objectively evidenced by HFUS [6,7].

## 2. Dermoscopy of Psoriasis Vulgaris

**Figure 1:**
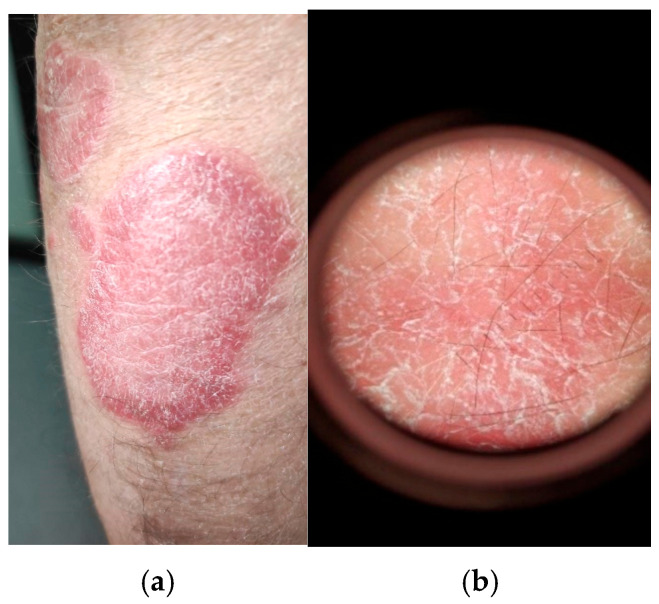
(**a**) Clinical examination of psoriasis vulgaris of a well-defined erythematous-to-scaly plaque, salmon-pink to bright red in color, covered with silvery-white, thick, multilayered, non-adherent scales; (**b**) conventional dermatoscopic examination (Dermatoscope Delta T ^®^, Heine Optotechnik GmbH & Co. KG, Gilching, Germany) showing silvery-white scales accompanied by dotted capillaries evenly distributed at the level of the plaque (own collection).

**Figure 2:**
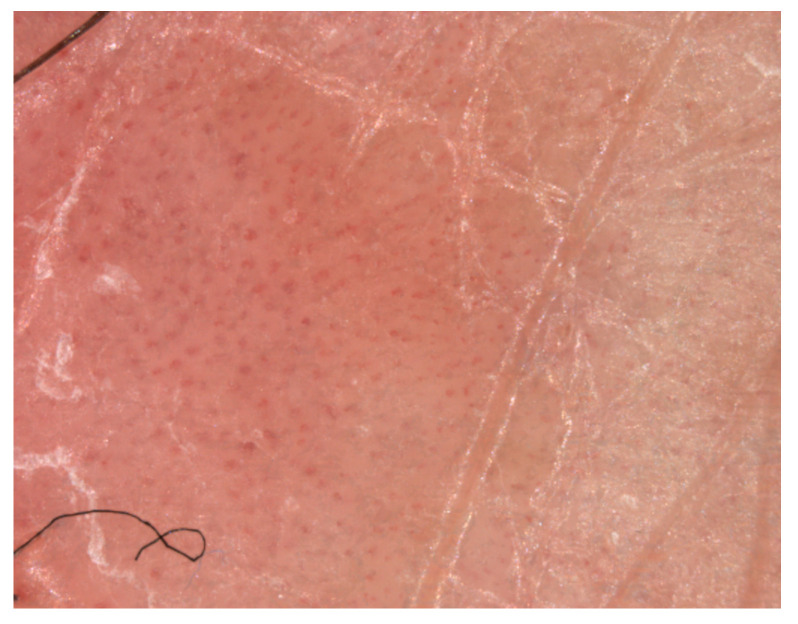
Visiomed microDERM AG^®^ (VISIOMED AG, Bielefeld, Germany) videodermatoscopy (×120 magnification) with visualization of dilated bushy or glomerular capillaries at the level of the psoriasis plaque (own collection).

Dermatoscopy is a non-invasive diagnostic imaging method that consists of the in vivo examination of mucocutaneous lesions, providing a detailed visualization of the pigmentary and structural characteristics of the epidermis, the dermo-epidermal junction, and the superficial and middle dermis. This technique is widely used in the diagnosis and evaluation of mucocutaneous lesions. Conventional dermatoscopy, with a magnification of 10×, highlights the vascular characteristics of psoriatic lesions in the form of punctate (Figure 1), globular or coiled (glomerular) vessels. In contrast, videodermatoscopy, with a magnification of up to 120×, allows the identification of dilated and elongated capillaries, with a “bushes” (Figure 2) or “basket-weave” appearance, arranged regularly and parallel to the skin surface. The vascular diameter is generally increased in the affected areas [8], the normal value of the capillary diameter being considered below 25 μm [9].

The dots and red blood cells, with a diameter of less than 0.1 mm, correspond to vascular loops arranged vertically within the elongated dermal papillae. On dermatoscopic examination, in psoriatic balanitis and inverse psoriasis, the lesions with minimally evident scales predominantly show a vascular type characterized by a regular pattern of well-demarcated red dots [5]. Under high-power digital dermoscopy (100–400×), capillaries appear dilated and elongated [10]. The identification of different vascular morphological types in dermatoscopic analysis, either manual or digital, may suggest a different diagnosis of psoriasis vulgaris [11]. Vazquez-Lopez et al. [12] described a distinct dermatoscopic vascular pattern in psoriasis vulgaris, characterized by the presence of red globular rings, a pattern that has good specificity for the diagnosis of psoriasis, but with low sensitivity [5,11,12]. The vascular pattern characterized by the presence of “red dots” can also be identified in other inflammatory conditions, such as lichen planus, porokeratosis, or pityriasis rosea. However, the distinctive feature in psoriasis vulgaris is the uniform distribution of these dermatoscopic structures over the entire surface of the lesion. After removal of the scales, the specific vascular pattern may be accompanied by punctate bleeding, a phenomenon known as the Auspitz sign [5]. In addition, the light red background and the presence of white scales are two additional dermatoscopic criteria characteristic of plaque psoriasis vulgaris.

The color of the scales is an important criterion in differentiating psoriasis vulgaris from other erythematous-squamous dermatoses. The presence of yellowish crusts is a strong negative predictor for the diagnosis of plaque psoriasis, orienting the diagnosis towards eczema [5]. Dermoscopic examination reveals specific characteristics for different skin conditions. In lichen planus, the presence of shiny white lines is observed; in pityriasis rosea, the scales are fine, white and located peripherally; in porokeratosis a characteristic squamous ring appears; purpuric pigmentary dermatoses are associated with red and brown pigmentary dots and globules; and in granulomatous conditions the orange color and the presence of thin vessels predominate [11].

Clinical and trichoscopic evaluations of the scalp are valuable tools in the diagnosis of psoriasis vulgaris, facilitating its differentiation from other inflammatory conditions, such as seborrheic dermatitis. In a study in which psoriasis was identified in 19% of participants, Rajkumar et al. [13] reported that trichoscopic examination of psoriatic lesions revealed the presence of red dots (92.3%), followed by silvery-white scales (84.6%), nonspecific erythematous areas (69.2%), and the “hidden hair” sign (61.5%). In trichoscopy, the “hidden hair” sign denotes a hair partially or completely covered by the adjacent skin or scales. In seborrheic dermatitis, the most frequently observed trichoscopic feature is perifollicular scaling (65.7%), followed by the presence of atypical vascular patterns (42.8%), yellow dots and interfollicular scaling (each with an incidence of 40%), the “hidden hair” sign (37.1%), nonspecific erythematous areas (34.2%), and red dots (8.5%) [13]. These results are consistent with those reported by Kibar et al., although a higher prevalence of atypical red vessels (67%) was observed in their cohort [14]. Depending on the affected anatomical area, psoriasis may present specific dermatoscopic features. Thus, in the scalp area, trichoscopy in psoriasis may indicate regular red dots, red blood cells and twisted red loops (in the case of videodermatoscopy) compared to seborrheic dermatitis in which atypical red vessels, arborized red lines and structural red areas can be observed. Differentiating psoriasis from seborrheic dermatitis can be difficult both clinically and dermatoscopically. However, the presence of twisted red loops is a more specific sign for psoriasis [14].

The importance of trichoscopy in the diagnosis of inflammatory scalp diseases was also highlighted by Waskiel-Burnat et al. [15], who identified the main trichoscopic features of psoriasis as silvery-white scaling, punctate glomerular vessels distributed regularly or in the form of twisted red loops, and punctate hemorrhages. In contrast, yellowish-white scaling and thin arborizing vessels are considered characteristic signs of seborrheic dermatitis [15].

Dermatoscopy of genital psoriasis reveals a series of specific features, largely similar to those observed in psoriasis localized to other skin regions, but with some particularities determined by the structure and anatomical conditions of the genital area. From a vascular point of view, the lesions present dotted vessels regularly and uniformly arranged on a homogeneous erythematous background, reflecting the dilation of capillaries in the dermal papillae. In some cases, tortuous capillaries with a bushy appearance can also be observed, indicating increased vascularization and intense inflammatory activity. Nonvascular elements include fine, silvery-white or whitish scales, located predominantly in the genital areas or in skin folds. However, the presence of scales may be reduced or even absent, as a result of the increased humidity and local friction characteristic of this region, over a light red background [16,17]. Dermatoscopy is of increased importance in the genital region, where clinical diagnosis can often be difficult due to the similarity to other dermatoses, such as lichen planus, lichen sclerosus or infectious balanitis. Examination difficulties such as contact with immersion gel, lighting and friction can influence the quality of dermoscopic images. Therefore, their interpretation should be performed in a complete clinical context, including the patient’s history, lesion location, and associated symptomatology, to reduce the risk of diagnostic error.

The dermatoscopic pattern characteristic of plaque, follicular, or guttate psoriasis in patients with dark skin phototypes (Fitzpatrick types V–VI) was analyzed in a systematic review [18]. The results revealed the presence of dotted vessels with a uniform or nonspecific distribution, associated with diffuse or patchy white scales. In addition, pigmentary structures, such as dots, globules, or brown, gray, or bluish astructural areas, have been described [18]. In the pediatric population of color with phototype V-VI, dermatoscopy tends to present with brown background shades or a higher prevalence of dark structural elements (brown, gray or blue dots, globules, blotches, clods or structureless areas), compared to the corresponding presentations observed in Caucasian populations [19]. Dermoscopic differences between light and dark skin reveal the predominant background colors and pigmentary changes that could be explained by the different intensity of skin pigmentation [20]. Dotted vessels can also be identified in psoriasis patients with darker skin phototypes, but they are usually less obvious compared to light skin phototypes. Due to the increased melanin content, the underlying erythema is partially masked, which causes the vessels to take on a purplish or red-brown hue, instead of the classic deep red appearance. In situations where vascular contrast is reduced, the use of polarized dermoscopy can facilitate the visualization of vascular structures by emphasizing chromatic differences. In situations where dermoscopy is difficult to interpret, HFUS represents a valuable complementary method in the evaluation of skin of color. HFUS allows visualization of the inflammatory process independent of the degree of skin pigmentation and highlights the thickening of the epidermis, the presence of a subepidermal hypoechoic band and the increase in dermal vascular signal, elements considered specific ultrasound indicators of psoriatic inflammation.

The presence of psoriatic arthropathy can be evidenced by the identification of dotted vessels in the dermoscopic analysis of the nail fold. Thus, early-stage psoriatic arthritis can be differentiated from seronegative rheumatoid arthritis by the detection of these dotted vessels in the nail fold, which contributes to increasing diagnostic specificity, especially in paucisymptomatic forms of the disease [21,22]. Dermoscopy is a useful tool in assessing the extent of lesions in nail psoriasis, allowing differentiation of matrix from nail bed involvement. Nail matrix involvement is characterized by the presence of deep pitting, leukonychia, and erythematous spots located at the lunula, while nail bed involvement is characterized by the appearance of the salmon spot sign (oil drop), splinter hemorrhages, and onycholysis [23,24,25]. Similarly, dermoscopy is a helpful method for differentiating from other nail conditions, such as traumatic onycholysis, onychomycosis, allergic contact dermatitis or those associated with the use of artificial nails [26]. Nail psoriasis presents specific dermatoscopic features, including deep pitting, salmon-colored macules, and a brownish band interposed between the area of onycholysis and the adjacent normal nail plate [27,28,29,30]. In contrast, traumatic onycholysis may show a line of separation of the nail plate from the nail bed that is regular, smooth, and delimited by apparently normal nail tissue [28,30]. When examined by dermatoscopy, onychomycosis may reveal jagged onycholytic edges, with whitish and sharp longitudinal indentations in the proximal portion of the onycholytic area, longitudinal bands on the surface of the nail plate accompanied by progressive color changes (from bluish-gray to blackish and later yellowish-whitish) [26,30,31]. Similarly, irregular distal endings with a pulverizing appearance, characteristic of the thickening of the nail plate may be observed in the total dystrophic form of onychomycosis [32,33]. Furthermore, Zhu et al. [32] developed a differential diagnostic model based on techniques, designed to distinguish between nail psoriasis, traumatic nail and onychomycosis. This model achieved an accuracy of 95.7%, a specificity of 98.8%, a sensitivity of 82.1%, and a Youden index of 0.809 [32].

The contribution to the assessment of therapy efficacy in patients with psoriasis vulgaris was investigated in a prospective, single-blind observational study [34] that included 101 patients diagnosed with psoriasis vulgaris and treated with systemic therapies, such as Acitretin, Methotrexate and biologic agents. Disease severity was assessed at baseline and at 1, 2, and 3 months using affected body surface area (BSA), Psoriasis Area and Severity Index (PASI) and Dermatology life Quality Index (DLQI) values. For monitoring, a target area of with minimal scaling was selected, which was photographed monthly and assessed by manual dermatoscopy and capillaroscopy. In patients who showed a favorable therapeutic response, a transition from capillary dilation to hemorrhagic spots or the absence of vascular structures was observed in the first month of treatment. A significant correlation was observed between the dermoscopic findings in the first month and the severity scores obtained at three months, assessed by PASI, BSA, and DLQI, that became stronger in the second and third months of follow-up. There were no significant differences between the groups treated with Acitretin, Methotrexate and biological agents (*p* > 0.05). Also, nail fold capillaroscopy performed at three months revealed significant differences compared to the initial assessment (*p* < 0.05). These results suggest that early use of dermatoscopic findings would allow the anticipation of therapeutic efficacy and the prevention of the application of ineffective treatments [34]. Dermoscopy may represent a useful tool for monitoring the evolution of skin lesions during therapy and, at the same time, for the early detection of relapses or adverse effects of topical treatment with topical steroids, such as the appearance of linear vessels at the level of the lesions, before they become clinically visible [12,35].

A study [36] evaluating the concordance between dermoscopic diagnosis with histopathology within inflammatory papulosquamous dermatoses reported, from 100 cases (of which 34 were psoriasis vulgaris), an overall positive correlation of 82%. For psoriasis cases, dermoscopy demonstrated a sensitivity of 94.1%, a specificity of 95.5%, and an accuracy of 95%, compared with an accuracy of 100% obtained by histopathological diagnosis. In psoriasis vulgaris lesions, the dermatoscopic appearance was characterized by an erythematous background, regularly arranged punctate vessels, patchy or in clusters, and diffuse or peripheral white scales. The authors concluded that dermoscopy is a valuable tool for differentiating inflammatory papulosquamous dermatoses and may reduce the need for skin biopsies, thus supporting its utility in clinical practice [36].

## 3. High-Frequency Ultrasonography of Psoriasis Vulgaris

HFUS is increasingly used in the evaluation of skin pathology, having multiple applications in the investigation of dermatological diseases. It is typically performed with probes with frequencies between 13.5 and 100 MHz, and the use of probes with fixed frequencies greater than 20 MHz, specific to HFUS, allows visualization of skin structures at a depth of approximately 6–8 mm [37]. Depending on the frequency of ultrasound, ultrasounds can penetrate a different number of surfaces and tissue interfaces. A higher frequency emitted by the transducer results in less penetration in depth, but provides a higher resolution of structures located closer to the probe [38]. For example, ultrasound performed with frequencies of 20–25 MHz allows visualization of both the skin and the subcutaneous tissue, while higher frequencies of 50–100 MHz provide a detailed image of only the epidermis [37]. A recently introduced technique uses variable frequencies between 6 and 18 MHz and a Doppler frequency between 4 and 7 MHz, allowing detailed characterization of the skin layers, deep structures, and vascular patterns in real time [38].

HFUS of normal skin shows a clear delimitation of the skin layers [39]. The ultrasound image of normal skin obtained by HFUS distinctly highlights three layers:

- The interface area, called entry echo, appears sonographically as an intensely echogenic band located at the surface of the skin. There is still debate regarding the correspondence of this band either with the entire thickness of the epidermis or exclusively with the epidermal stratum corneum. Camerota et al. argue that entry echo is determined by the difference in acoustic impedance between the ultrasound gel and the skin [40]. According to Ximena Wortsman, the epidermis is represented sonographically by a hyperechoic line with variable heterogeneity, due to the increased keratin content in this layer. In the palmar-plantar area, where the skin is thicker, the epidermis presents a bilaminar hyperechoic appearance. Structures such as melanocytes, melanin, Langerhans cells or Merkel cells cannot be accurately identified by ultrasound. The average thickness of the epidermis in healthy individuals is approximately 0.6 mm, varying between 0.1 and 0.8 mm [38].
- The dermis appears sonographically as a hyperechoic structure, less shiny than the epidermis, and has a heterogeneous echogenicity. The papillary dermis is visible as a thin hypoechoic band located immediately beneath the epidermis, having a lower echogenicity than the reticular dermis. This difference is attributed to the more compact organization of the thick collagen bundles in the reticular dermis, located deeper, as well as the poorly represented vascularity on color Doppler examination [40]. The reticular dermis appears sonographically as a thicker, slightly hyperechoic band immediately beneath the papillary dermis, due to its high collagen content, and varies in thickness from approximately 1 to 2.5 mm [40], depending on the anatomical region—thinner in the forearms and thicker in the lumbar region. On color Doppler ultrasound, the reticular dermis usually shows no or minimal blood flow signals, depending on the area being examined.
- The subcutaneous tissue appears ultrasonographically as a hypoechoic structure, determined by the presence of adipose lobules, with hyperechoic areas corresponding to fibrous septa. Examination by color Doppler ultrasound reveals blood vessels with reduced flow [38]. Hair follicles appear as hypoechoic dermal bands (“shadows”), wider at the bottom, parallel to each other and oriented obliquely, necessitating avoidance of longitudinal transducer positioning for optimal visualization.

**Figure 3:**
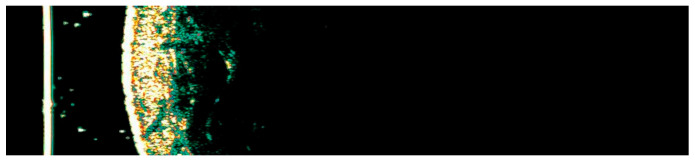
HFUS analysis with Dermascan C USB^®^ 20 MHz B-mode (Cortex Technology ApS, Hadsund, Denmark) of normal skin adjacent to a psoriasis plaque showing the epidermis as an echogenic band at the surface of the skin, the dermis, which is hyperechoic, less shiny and is characterized by heterogeneous echogenicity and the hypodermis which is hypoechoic with an appearance generated by fat lobules (own collection).

**Figure 4:**
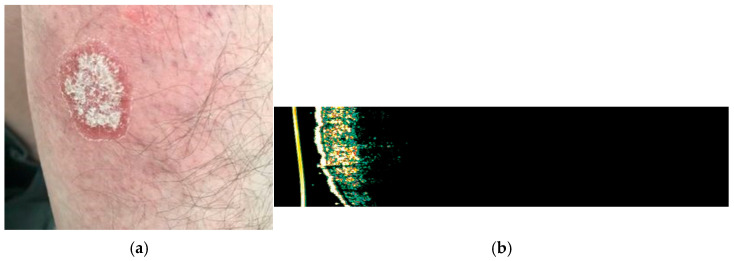
(**a**) Clinical appearance of erythematous-squamous plaque psoriasis on the right forearm; (**b**) HFUS analysis with Dermascan C USB^®^ (20 MHz B-mode) of the examined psoriasis plaque showing thickening of epidermis with a hyperechogenic band, which represents hyperkeratosis and parakeratosis accompanied by hypoechogenic band, corresponding to the elongation of the dermal papillae.

In the skin lesions characteristic of psoriasis vulgaris, epidermal and dermal thickening is observed, determined by keratinocyte proliferation and inflammatory infiltrate with a homogeneous hyperechoic appearance and a hypoechogenic subepidermal band. The dermis is thickened and hypoechoic compared to the perilesional areas (Figure 3 and Figure 4), showing increased blood flow when evaluated by color Doppler ultrasound. In addition, the total skin thickness is increased in psoriatic plaques, and its decrease during therapy can be objectively monitored by HFUS [6,7].

**Figure 5:**
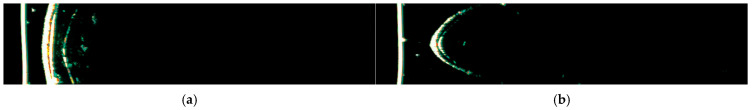
(**a**) HFUS examination (Dermascan C^®^ 20 MHz) of a thumb healthy nail unit: longitudinal section of the right thumb nail apparatus indicates the nail blade with bilaminar aspect, the nail bed and the bone plane (distal phalanx); (**b**) HFUS examination in transverse section shows the nail blade with bilaminar aspect, the nail bed and the bone plane distal phalanx (own collection).

The healthy nail unit is composed of several anatomical structures, the nail bed, the nail plate, the periungual fold, the distal interphalangeal joint and the distal tendon insertion (transverse and longitudinal). The nail plate appears sonographically as a hyperechoic structure, highlighting two parallel hyperechoic lines corresponding to the ventral and dorsal plates, separated by a hypoechoic zone. The nail bed and the nail matrix are hypoechoic, the matrix being slightly more hypoechoic than the proximal bed, creating an obvious contrast with the hyperechoicity of the overlying dermis (Figure 5).

The periungual region includes the proximal and lateral nail folds. The skin layers in these regions have echogenicities comparable to those of normal skin elsewhere in the body. In adults, the nail bed varies in thickness from 0.7 to 6 mm, and from 0.3 to 0.65 mm. Color Doppler ultrasound reveals a pattern of reduced blood flow, especially near the bony margin of the distal phalanx [7,38].

**Figure 6:**
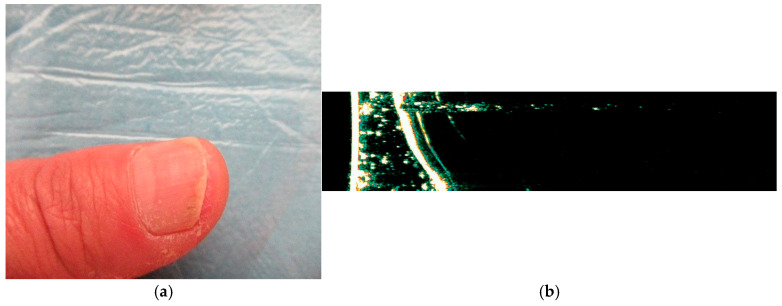
(**a**) Clinical appearance of psoriatic onychopathy of the right thumb with longitudinal striations and leukonychia; (**b**) HFUS examination (Dermascan C^®^ 20 MHz) of the right thumb indicates the bilaminar nail plate which is thickened and has hyperechoic irregularities, with nail bed changes as thickening and the bone plane of the distal phalanx (own collection).

Nail psoriasis is diagnosed clinically by identifying characteristic signs, and the diagnosis can be completed by ultrasonography. This imaging method allows for the objective evaluation of nail changes from the early to the late stages of the disease. Ultrasound can reveal thickening over 2 mm (corresponding to an increased distance between the ventral plate and the bony edge of the distal phalanx), as well as decreased echogenicity of the nail bed, accompanied by loss of definition of the ventral plates, a phenomenon attributed to nail bed hyperkeratosis, which can be subclinical in the early stages (Figure 6). The dorsal nail plates may present ultrasound changes, including focal hyperechoic deposits and areas with a wavy appearance in late forms of nail psoriasis [7,38]. Hypervascularization detected by color Doppler ultrasound is manifested in the active phase of the disease at the proximal and distal levels, whereas in the chronic phase it predominates distally. Early signs of psoriatic onychopathy include loss of bimorphic appearance, presence of nail ridges, and nail plate surface irregularities [41]. Moreover, ultrasound allows assessment of concomitant enthesopathy of the digital extensor tendon in the distal interphalangeal joint.

Inflammatory involvement in plaque psoriasis vulgaris can also target joints and entheses. The anatomical connection between the last phalanx and the nail unit determines the correlation between psoriatic arthritis and nail changes [42]. When ultrasonographic examination is performed by a radiologist, the nail apparatus is frequently evaluated concurrently with the distal interphalangeal joint, given their close anatomical relationship. Psoriatic arthritis is a chronic systemic inflammatory condition, characterized by inflammatory joint involvement, with an estimated prevalence between 0.05% and 0.25% in the general population and reported in 6–41% of patients diagnosed with psoriasis [43]. Cutaneous psoriasis may precede the onset of psoriatic arthritis in 85% of cases [44]. At the joint level, hypertrophied synovial membranes, anechoic joint fluid and periarticular erosions may be seen in ultrasound examination, most commonly located at the interphalangeal joints. Tendinopathy, characterized by hypoechoic or heterogeneous echogenicity, is observed especially at the entheses and has been reported ultrasound-guided even in patients with psoriasis in subclinical stages of the disease. In active forms, color Doppler imaging can detect increased blood flow in the synovium, indicative of inflammation [38]. To evaluate the value of HFUS in identifying subclinical psoriatic arthropathy, the authors of a cross-sectional study [44] included a group of 117 patients diagnosed with plaque psoriasis vulgaris, without clinical manifestations of joint damage. The ultrasound evaluation targeted several parameters, including synovial thickening, the presence of bone erosions, structural changes in tendons (thickening and hypoechogenicity), the presence of calcifications, as well as vascular changes detected by color Doppler examination. The results of the study revealed that the distal interphalangeal joint and the Achilles tendon were most frequently involved, with synovial thickening at the distal interphalangeal joint observed in 57% of patients and Achilles tendon thickening in 33% of the cases investigated. The authors concluded that HFUS is a valuable tool for detecting subclinical synovitis or enthesitis in asymptomatic patients and the distal interphalangeal joint and Achilles tendon can be used as useful landmarks in the ultrasound screening of subclinical psoriatic arthropathy [44]. Early diagnosis of psoriatic arthritis is essential for prognosis, as disease progression often leads to irreversible joint destruction and loss of function.

HFUS of the nails may be useful for monitoring therapeutic response, as it allows for the detection of reductions in nail plate thickness [45]. Michelucci et al. [46] investigated the associated changes using HFUS at frequencies of 70–100 MHz, monitoring both the nail bed and matrix during monoclonal antibody (mAb) therapy. The study included 10 patients with psoriatic onychopathy, who were evaluated clinically and sonographically at baseline, as well as 1 and 3 months after the initiation of mAb therapy. Several ultrasound parameters were assessed, including nail plate thickness, nail bed thickness, nail insertion length, nail matrix length, and nail matrix thickness. The results demonstrated a statistically significant reduction (*p* < 0.05), and after one month of initiating biological therapy with anti-TNF-α and anti-IL monoclonal antibodies, a decrease in nail plate thickness was observed even before clinical improvement was detectable. These findings suggest that it may represent an effective imaging method for monitoring psoriatic onychopathy, revealing subclinical signs of early response to treatment [46,47]. In addition, psoriatic onychopathy was associated with the presence of enthesopathy, even in the absence of obvious clinical signs of the disease [48].

In psoriasis vulgaris, objective parameters of response to therapy assessed by ultrasound include reduction in epidermal and dermal thickness, as well as disappearance of the hypoechoic subepidermal band. This hypoechoic band may also be present in other inflammatory conditions, such as atopic dermatitis or contact dermatitis [49]. Dermal thickness measured by ultrasound correlates with disease severity assessed by the PASI score [41,50]. HFUS may provide objective and characteristic information, inaccessible to clinical assessment that can be used for a detailed assessment of psoriasis vulgaris plaques, as well as for monitoring disease activity and response to topical and/or systemic treatments. The importance of ultrasound monitoring of psoriasis activity and severity has been demonstrated in several studies [9,51]. HFUS of the skin or nail apparatus can be used to assess the response to topical and/or systemic treatments. In a pilot study, Lacarrubba et al. [52] evaluated 30 patients with psoriasis vulgaris, compared with 10 healthy volunteers, after application of topical foam of clobetasol propionate 0.05%. Monitoring of the response to treatment by 20 MHz HFUS demonstrated a reduction in the thickness of the psoriatic plaque, equaling the values to those of the adjacent healthy skin in all treated plaques. Equal values of skin thickness were obtained with those of adjacent healthy skin, in all treated plaques, without differences between the two groups of psoriasis patients. In the group of healthy volunteers, no ultrasound variations in skin thickness were detected at the end of the study [52].

In a short-term follow-up study of patients treated with Etanercept [41], clinical parameters assessed by the PASI score, the Doppler ultrasound vascular aspects obtained at frequencies of 7–14 MHz and histological changes were compared in a group of 12 patients. The study revealed a positive correlation between Doppler scores, PASI scores and histological vascular changes. The authors emphasize the need for studies on a larger sample to reliably assess clinical and imaging activity of the disease and to appreciate the therapeutic efficacy of different biological agents [41].

A multimodal clinical trial, combining ultrasonographic and videodermatoscopic analysis, monitored patients diagnosed with psoriasis vulgaris treated with biologic agents (Adalimumab, Etanercept and Ustekinumab). Results showed mean reductions in the appearance of bushy capillaries, detected by videodermatoscopy, of 73.5%, 49.7%, and 66.4%, respectively. Ultrasound assessment of psoriatic plaques revealed reductions of 33.5% at 15 days, 63.6% at 30 days, and 79.3% at 60 days in patients treated with Etanercept [9]. After 60 days of treatment, 23 of the 42 plaques analyzed showed complete normalization. In patients treated with Etanercept, 14 target plaques showed normalization of the vascular pattern, while two plaques maintained a bushy vascular pattern, despite achieving clinical remission with a TLS score equal to 0. In conclusion, it proved to be the parameter with the most pronounced improvement in monitoring therapeutic response [9].

Dermatoscopic and ultrasound monitoring of the efficacy of biological therapy in patients with moderate to severe psoriasis was investigated by Wang et al. [53]. The study followed the correlation between clinical, dermatoscopic and ultrasound assessments. Dermoscopic examination, performed by a digital system (MoleMax HD video dermatoscope), assessed the erythematous background, vascular morphology and the presence of scales, using a 4-point scale. Ultrasound analysis, performed with 20 and 50 MHz probes, determined the thickness of the superficial hyperechoic band and the subepidermal hypoechoic band. In total, 24 of 27 patients were included in the final analysis, by comparing clinical, dermatoscopic and data obtained by ultrasound, before and after treatment with biological agents (Adalimumab, Secukinumab and Ixekizumab). In the dermoscopic assessment, the scores for erythematous background, vessels, and scales were reduced by 78.5%, 84.1%, and 86.5%, respectively. Ultrasound analysis revealed a mean reduction of 53.9% in the thickness of the superficial hyperechoic band and 89.9% in the thickness of the hypoechoic subepidermal band (SLEB). The most significant decreases in clinical and imaging variables—TLS, dermatoscopic scale score, and SLEB thickness were recorded at week 4, with reductions of 55.4%, 57.7%, and 59.1%, respectively (*p* > 0.05). Strong correlations were found between SLEB thickness and the scores for vessels and erythematous background, as well as between the thickness of the superficial hyperechoic band and scales. These results confirm the usefulness of combining dermatoscopy with HFUS in monitoring therapeutic response in patients with moderate to severe plaque psoriasis [53].

A comparative analysis of imaging features obtained by dermatoscopy and HFUS in psoriasis is presented in Table 1.

Similarly, Table 2 offers a comparative description of dermoscopy and HFUS in psoriasis monitoring.

## 4. Conclusions

Dermatoscopy and high-frequency skin ultrasound are valuable tools in optimizing the management of patients with psoriasis, contributing both to the establishment of an early diagnosis and to the objective monitoring of the evolution of lesions under treatment, including in areas with anatomical particularities, such as the facial, palmo-plantar or genital regions. Videodermatoscopy is a complementary imaging method for therapeutic monitoring, highlighting the fact that, in certain cases, the persistence of the vascular pattern characterized by dilated, bushy capillaries at the level of psoriatic plaques is maintained even in the context of partial or complete clinical resolution, suggesting continued disease activity and the need for prolonged therapy.

HFUS plays an important role in the assessment of therapeutic response in patients with psoriasis. The first detectable change under topical and/or systemic treatment at the level of target plaques is represented by the reduction in skin thickness and the hypoechoic subepidermal band. The main objective indicators of therapeutic efficacy, evaluated by HFUS, are the decrease in epidermal and dermal thickness, as well as the disappearance of the hypoechoic band. In nail psoriasis, often associated with arthropathic forms, the use of imaging techniques is essential for the diagnosis of subclinical forms and for monitoring disease progression. Ultrasound determination of nail bed and blade thickness in patients with psoriatic onychopathy can be a valuable marker for both diagnosis and assessment of therapeutic response.

Continuing imaging research is indispensable, since the integration of these methods into clinical practice can optimize the management of patients with inflammatory diseases and contribute to improving their quality of life.

## Figures and Tables

**Table 1:** Dermoscopy and HFUS advantages and limitations in psoriasis examination.

Examination Method	Advantages	Limitations
Dermoscopy	- noninvasive imaging technique - improves the diagnostic accuracy of psoriasis and allows differentiation from other inflammatory dermatoses (visualization of vascular pattern: dotted, globular vessels with uniform distribution, and scaling) - easy use of the instrument - low costs - observation of lesions located in anatomical places difficult to examine by using special lenses - useful for monitoring the response to topical and systemic treatment, by evaluating morphological and vascular changes [9,11,52,53]	- it provides limited information at the level of the epidermis and superficial dermis, without allowing the evaluation of deep skin structures - it does not allow the assessment of subclinical inflammation nor the determination of dermal thickness, due to the limitation of the penetration depth of the method (approximately 200–250 μm) [7] - in the case of manual dermoscopy, the light intensity cannot be adjusted, which may limit optimal visualization of certain skin structures; - in the case of digital dermoscopy, its use may be limited by the increased time required for the investigation, high costs and equipment - the results may be influenced by the degree of skin pigmentation (vascular patterns such as dotted vessels may be less obvious, with brown background shades due to increased melanin content) [20]
High-Frequency Ultrasound (HFUS)	- is a real-time noninvasive imaging examination - provides a clear delineation of skin layers (homogeneous thickening of the epidermis that appears as a hyperechogenic band, with the presence of a hypoechogenic subepidermal band and thickening of the dermis), without presenting significant limitations on the depth of signal penetration; - may offer quantitative and qualitative analysis of blood flow (increased dermal vascularity) [7] - it allows obtaining objective and quantifiable data, such as measuring skin thickness and determining the echogenicity of skin structures - useful in monitoring response to therapy (the effectiveness indicators of therapy are the decrease in epidermal and dermal thickness and the disappearance of the hypoechoic band) - the performance of the method cannot be affected by variations in skin pigmentation [9,51]	- is dependent on the operator’s experience - need of specialized equipment - time consuming (may require a longer examination time than dermoscopy) - the hyperkeratosis could show a potent posterior acoustic shadowing artifact - does not highlight certain morphological characteristics of the skin surface, such as scaling or the presence of pinpoint bleeding - it is necessary to avoid compression to increase the accuracy of the results - an optimal setting of the B-mode scan is necessary, with correct signal amplification/gain setting - errors may occur related to incorrect adjustments of contrast, brightness and focus of Doppler technical parameters [7]
Combined (Dermoscopy and HFUS)	- can provide objective markers (surface elements—vascular pattern and scaling in the case of dermatoscopy and objective elements regarding the thickness of psoriasis plaques/nail apparatus) (HFUS) with a complementary role in increasing the accuracy of diagnosis and staging of the disease; - allows the assessment of the response to therapy by evaluating the vascular pattern (bushy capillaries) and the morphology of the lesions (thickness of the epidermis and dermis, disappearance of the subepidermal hypoechoic band) [9] - in patients of color, when dermatoscopy does not provide sufficient vascular elements in difficult-to-analyze cases, HFUS can provide additional morphological diagnostic elements, not influenced by skin color [7]	- examination time is longer; - requires specialized equipment and advanced technical medical training; - availability may be limited in routine clinical practice.

**Table 2:** Dermoscopy and HFUS characteristics in psoriasis monitoring.

Examination Parameters	Dermoscopy	HFUS	Therapeutic Response	Psoriasis or Clinical Evolution
Vascular features	- punctate, globular or coiled vessels, “bushes” or “basket-weave” capillaries, regularly and parallel arranged to the skin surface [8,9]	- increased flow in the dermis visible with Doppler in psoriatic plaques - increased flow in the nail bed in psoriatic onicopathy	- transition from capillary dilation to hemorrhagic spots or the absence of vascular structures after systemic therapy with Acitretin, Methotrexate and biologic agents (anti-TNF alfa, anti-IL 17) or topical corticosteroids (effective treatment) or persistence (poor treatment response) [34,53]	- the density and caliber of cutaneous vessels correlate significantly with the degree of inflammatory activity and the thickness of the psoriatic plaque [9]
Hyperkeratosis and surface features	- presence of white scales on a light red background; sometimes the presence of punctate bleeding, a phenomenon known as the Auspitz sign [5]	- the epidermis appears as a hyperechoic line with variable heterogeneity, due to the increased keratin content in this layer [38]	- severe keratinocyte proliferation may be correlated with severity disease score such as dermatoscopic scale score or Target Lesion Score [34,53]	- reduction in scaling and reflectivity after keratolitic therapy
Epidermis and dermis thickness and/or nail plate/nail bed thickness	- identification of dotted vessels in nail fold suggests psoriatic arthropathy - allows differentiation of nail matrix involvement (presence of deep pitting, leukonychia, and erythematous spots located at the lunula) from nail bed involvement (appearance of the salmon spot sign -oil drop), splinter hemorrhages, and onycholysis [23,24,25]	- dermo-epidermal thickening - the dermis is thickened and hypoechoic compared to the perilesional areas - appearance of hypoechoic band in the superficial dermis that correlates with inflammation (Subepidermal Low- Echogenic Band) [6,7] - thickening over 2 mm of nail bed and nail plate, decreased echogenicity of the nail bed, accompanied by loss of definition of the ventral plates, - the dorsal nail plates may present focal hyperechoic deposits, areas with a wavy appearance [7,38,41]	- reducing the thickness of psoriasis plaque equaling the values to those of the adjacent healthy skin after topical therapy with corticosteroids [52], calcipotriol and/or systemic therapy, phototherapy - nail plate and nail bed thickness reduction, after biological therapy with anti-TNF-α, anti-IL monoclonal antibodies [45,46,47] - psoriatic onychopathy was associated with the presence of enthesopathy, even in the absence of obvious clinical signs of the disease [48]	- epidermal and dermis thickening is an imaging indicator of a more severe form of the disease correlating with histology - a decrease in nail plate thickness may be observed even before clinical improvement is detectable [46,47,48]

## Abbreviations

The following abbreviations are used in this manuscript:

HFUS	High-frequency cutaneous ultrasonography
TNF-alpha	Tumor Necrosis Factor alpha
BSA	Body Surface Area
PASI	Psoriasis Area and Severity Index
DLQI	Dermatology life Quality Index
MHz	Megahertz
SLEB	Subepidermal low echogenic band
TLS	Target Lesion Skin
